# Supplementary material for: A large-scale multi-ancestry genome-wide association study of chronic prostatitis/chronic pelvic pain syndrome in men
Source: Nat Commun. 2026 Jan 9;17:343. doi: 10.1038/s41467-025-64954-2 (PMC12789528; doi:10.1038/s41467-025-64954-2)
Supplement: Supplementary file 2 — Description of Additional Supplementary Files [file 41467_2025_64954_MOESM2_ESM.pdf]

**Description of Additional Supplementary Files**

Supplementary Data 1: ST1. Multip Ancestry Genome Wide Signficicant Loci and FUMA genes

Supplementary Data 2: ST2. MAGMA mapped genes

Supplementary Data 3: ST3: Annotation of regulatory genetic variants

Supplementary Data 4: ST4. FUMA aggregated tissue expression

Supplementary Data 5: ST5. Gene-set analysis results

Supplementary Data 6: ST6. GWAS catalog annotation for significant SNPs

Supplementary Data 7: ST7. Genomic correlation between CP/CPPS and all phenotypes available on Complex-Traits Genetics Virtual Lab

Supplementary Data 8: ST8. Bivariate MiXeR analysis of CPCPPS, BPH, and CaP.

Supplementary Data 9: ST9. Mendelian randomization results from MR base

Supplementary Data 10: ST10. Mendelian randomization results from CAUSE

Supplementary Data 11: ST11. Case-Case GWAS genes.

Supplementary Data 12: ST12. Enrichr- enriched pathways from CP/CPPS network
