# Supplementary material for: A large-scale multi-ancestry genome-wide association study of chronic prostatitis/chronic pelvic pain syndrome in men
Source: Nat Commun. 2026 Jan 9;17:343. doi: 10.1038/s41467-025-64954-2 (PMC12789528; doi:10.1038/s41467-025-64954-2)
Supplement: Supplementary file 15 — Reporting Summary [file 41467_2025_64954_MOESM15_ESM.pdf]

nature portfolio

Corresponding author(s): Marianna Gasperi

Last updated by author(s): 05/29/20205

Reporting Summary

Nature Portfolio wishes to improve the reproducibility of the work that we publish. This form provides structure for consistency and transparency in reporting. For further information on Nature Portfolio policies, see our Editorial Policies and the Editorial Policy Checklist.

Statistics

For all statistical analyses, confirm that the following items are present in the figure legend, table legend, main text, or Methods section.

n/a	Confirmed	
☐	☒	The exact sample size (*n*) for each experimental group/condition, given as a discrete number and unit of measurement
☐	☒	A statement on whether measurements were taken from distinct samples or whether the same sample was measured repeatedly
☒	☐	The statistical test(s) used AND whether they are one- or two-sided<br>*Only common tests should be described solely by name; describe more complex techniques in the Methods section.*
☐	☒	A description of all covariates tested
☐	☒	A description of any assumptions or corrections, such as tests of normality and adjustment for multiple comparisons
☐	☒	A full description of the statistical parameters including central tendency (e.g. means) or other basic estimates (e.g. regression coefficient) AND variation (e.g. standard deviation) or associated estimates of uncertainty (e.g. confidence intervals)
☐	☒	For null hypothesis testing, the test statistic (e.g. *F*, *t*, *r*) with confidence intervals, effect sizes, degrees of freedom and *P* value noted<br>*Give P values as exact values whenever suitable.*
☒	☐	For Bayesian analysis, information on the choice of priors and Markov chain Monte Carlo settings
☒	☐	For hierarchical and complex designs, identification of the appropriate level for tests and full reporting of outcomes
☐	☒	Estimates of effect sizes (e.g. Cohen's *d*, Pearson's *r*), indicating how they were calculated

Our web collection on statistics for biologists contains articles on many of the points above.

Software and code

Policy information about availability of computer code

Data collection: No specific software was used for data collection in this study

Data analysis: MVP samples were genotyped using a 723,305-SNP Affymetrix Axiom Biobank array, customized to include variants of interest in multiple diverse ancestries. 10 Imputation was performed with Minimac4 using data from the TopMed reference panel. Analyses were performed using MVP Release 4 data (GRCh38). Final genotype data consisted of 96 million variants. Principal components were calculated for each ancestry using PLINK 2.0 alpha. We used Harmonizing Genetic Ancestry and Self-identified Race/Ethnicity (HARE) groups to define race/ethnicity. GWAS analysis was carried out separately for CP/CPPS in EUR, AFR, and HIS. Logistic regression analyses were performed to test association between phenotypes and imputed dosages using REGENIE v3.1.3. GWAS was performed on populations stratified by ancestry. The model included the first 10 principal components of genotype as covariates. SNPs with imputation INFO scores > 0.3, minor allele frequency (MAF) ≥ 0.01, and HWE > 1 x 10-15 were reported. A genome-wide significance (GWS) was set for the primary analysis as P ≤ 5.0 x 10-8. Meta-analyses were conducted across summary statistics from 3 ancestries (EUR, AFR, and HIS) using Plink and MR-MEGA, with default parameters. SNPs were annotated to nearby and relevant genes using the FUMA tool, positional mapping, and eQTL mapping from all current eQTL databases available on FUMA. We used the MAGMA tool, analyzed through the FUMA webtool, for gene-based, gene-pathway, and tissue enrichment analyses. Tissue-specific enrichment analysis on the genes mapped to marginally significant EUR loci (p<5x10-6) was run using the TSEA tool. SNPs with potential regulatory relationships were further annotated through regulomeDB. Linkage disequilibrium score regression (LDSC) was used to estimate SNP-based heritability of CP/CPPS and correlations with other traits. To further explore the relationship between CP/CPPS and other phenotypes, we leveraged publicly available GWAS data within the Complex Trait Genetics Virtual Lab. Mendelian randomization (MR) was performed using the R package associated with MRbase, twoSampleMR and whole-GWAS CAUSE. We employed the mixed effects score regression (MiXeR) framework to estimate genetic overlap between CP/CPPS and CaP and between CP/CPPS and BPH. To further investigate shared genetic architecture between CP/CPS, CaP, and BPH, we utilized a cross-case GWAS (CC-GWAS) approach. We employed a network propagation algorithm to connect the cross-ancestry high-confidence genes (genes annotated to p<5 x10-8 loci) to any

interacting medium-confidence genes (genes annotated to p<5 x10-6 cross-ancestry loci), thus, boosting the signal from any marginal results. The network propagation was seeded with the CP/CPPS high-confidence FUMA-mapped genes using the STRING high-confidence interactome (v12). Highly significantly interacting genes (z>4) were retained for further analysis. Functional enrichment analysis was calculated with enrichR.

For manuscripts utilizing custom algorithms or software that are central to the research but not yet described in published literature, software must be made available to editors and reviewers. We strongly encourage code deposition in a community repository (e.g. GitHub). See the Nature Portfolio guidelines for submitting code & software for further information.

Data

Policy information about availability of data

All manuscripts must include a data availability statement. This statement should provide the following information, where applicable:

- Accession codes, unique identifiers, or web links for publicly available datasets
- A description of any restrictions on data availability
- For clinical datasets or third party data, please ensure that the statement adheres to our policy

Raw MVP data are protected and are not available due to privacy reasons. GWAS and meta-analysis summary statistics will be available in dbGaP (https://www.ncbi.nlm.nih.gov/gap/) upon publication under accession phs001672. MVP summary data access can be obtained by submitting a data access request through dbGaP.

Research involving human participants, their data, or biological material

Policy information about studies with human participants or human data. See also policy information about sex, gender (identity/presentation), and sexual orientation and race, ethnicity and racism.

Reporting on sex and gender

Findings apply only to men. Only individuals with self-reported gender as men were used in the analyses. Sex or gender differences were not considered because chronic prostatitis/chronic pelvic pain syndrome (CP/CPPS) is a male urinary condition.

Reporting on race, ethnicity, or other socially relevant groupings

Race, ethnicity, and ancestry were considered in the study design.
We used Harmonizing Genetic Ancestry and Self-identified Race/Ethnicity (HARE) groups to define race/ethnicity. Briefly, HARE enhances classification by integrating self-identified race/ethnicity (SIRE) and genetically inferred ancestry (GIA). HARE ensures accurate classification using GIA to refine and, if necessary, impute SIRE, improving reliability of race/ethnicity assignment in genetic research. Less than 2% of individuals are not assigned a HARE group when participant-identified and genetically inferred ancestry data produce discordant results. Here, we use the term “Hispanic” for the HARE race and ethnicity groups comprised of individuals who are Latino or Hispanic, the term “European” for individuals who are White but not Hispanic, and “African” for individuals who are Black but not Hispanic.
People of East Asian and South Asian ancestry were not analyzed due to the low numbers in MVP.

A total of 583,395 participants had available phenotype and genotype information and were used for GWAS analysis (Table 1). The HARE sample sizes for European ancestry (EUR, n=430,306 total; 10,035 cases, 420,271 controls), African ancestry (AFR, n=106,081 total; 3,553 cases, 102,528 controls), and Hispanic ethnicity (HIS, n=47,008 total; 987 cases, 46,021 controls) groups were used in this analysis. People of East Asian and South Asian ancestry were not analyzed due to the low numbers of Asian individuals in MVP.

Population characteristics

The sample included 583,395 participants with 14,575 CP/CPPS cases and 568,820 controls from three ancestral backgrounds, resulting in an overall prevalence of 2.50%. Table 1 shows the average age and CP/CPPS prevalence by HARE-based categories.

Recruitment

With the 2022 MVP data release, 819,417 participants were enrolled in the MVP and 662,681 had genetic data available for analyses reported here. MVP data includes self-reported survey, electronic health record (EHR), and genetic data.

Ethics oversight

The VA cIRB and the Research and Development Committee at VA San Diego Healthcare System and VA Puget Sound System approved the current analyses. Details on the cohorts and phenotypes are provided in the supplement.

Note that full information on the approval of the study protocol must also be provided in the manuscript.

Field-specific reporting

Please select the one below that is the best fit for your research. If you are not sure, read the appropriate sections before making your selection.

☒ Life sciences ☐ Behavioural & social sciences ☐ Ecological, evolutionary & environmental sciences

For a reference copy of the document with all sections, see nature.com/documents/nr-reporting-summary-flat.pdf

Life sciences study design

All studies must disclose on these points even when the disclosure is negative.

Sample size	Sample size was not predetermined but reflects the results of the phenotyping effort of individuals with CP/CPPS in MVP men with genetic data.
Data exclusions	Data exclusions were performed based on either failure of predetermined data quality control or planned phenotype exclusions to ensure the validity of case/control criteria. Individuals observed to have low genotyping quality were excluded. Ancestries other than European, African, Hispanic/LatinX were excluded due to insufficient sample size for meaningful analysis in the currently available data. For phenotype-based exclusions, individuals with insufficient EHR data, women, missing fender, no genetic information, or missing ancestry were excluded, as detailed in the methods. The metrics used as exclusion criteria were established before analysis, but some thresholds used (e.g., cutoffs for ancestry analysis for strata) were evaluated during the QC process. All the above exclusions were made in accordance with the planned study protocol and are detailed in the methods section.
Replication	We attempted cross-ancestry replication of the genome-wide significant loci in the study. Results were mixed, with some loci replicating and some trending towards significance. As we note in our results, the smaller ancestry cohorts were potentially underpowered. No external sources of CP/CPPS GWAS were available during analyses. A sample from FinnGen (https://www.finngen.fi/en) trait called "Inflammatory disease of the prostate (prostatitis)" was used as the best available analog, though CP/CPPS is a distinct phenotype. In the summary statistics for this trait, the lead SNPs for two out of three CP/CPPS genome-wide significant loci replicated at nominal significance. The genome-wide significant SNPs from the MVP CP/CPPS cross-ancestry analysis did not replicate in FinnGen (p>0.05).
Randomization	Randomization of experimental groups was not applicable to this study. Our study assesses the observed association between teh natural randomization of genotype and the ascertained phenotype of CP/CPPS.
Blinding	Blinding was not applicable to this study.

Reporting for specific materials, systems and methods

We require information from authors about some types of materials, experimental systems and methods used in many studies. Here, indicate whether each material, system or method listed is relevant to your study. If you are not sure if a list item applies to your research, read the appropriate section before selecting a response.

Materials & experimental systems

n/a	Involved in the study	
☒	☐	Antibodies
☒	☐	Eukaryotic cell lines
☒	☐	Palaeontology and archaeology
☒	☐	Animals and other organisms
☒	☐	Clinical data
☒	☐	Dual use research of concern
☒	☐	Plants

Methods

n/a	Involved in the study	
☒	☐	ChIP-seq
☒	☐	Flow cytometry
☒	☐	MRI-based neuroimaging

Plants

Seed stocks	*Report on the source of all seed stocks or other plant material used. If applicable, state the seed stock centre and catalogue number. If plant specimens were collected from the field, describe the collection location, date and sampling procedures.*
Novel plant genotypes	*Describe the methods by which all novel plant genotypes were produced. This includes those generated by transgenic approaches, gene editing, chemical/radiation-based mutagenesis and hybridization. For transgenic lines, describe the transformation method, the number of independent lines analyzed and the generation upon which experiments were performed. For gene-edited lines, describe the editor used, the endogenous sequence targeted for editing, the targeting guide RNA sequence (if applicable) and how the editor was applied.*
Authentication	*Describe any authentication procedures for each seed stock used or novel genotype generated. Describe any experiments used to assess the effect of a mutation and, where applicable, how potential secondary effects (e.g. second site T-DNA insertions, mosiacism, off-target gene editing) were examined.*
